# Concerning a Work Movement Velocity Action Level Proposed in “Action Levels for the Prevention of Work-Related Musculoskeletal Disorders in the Neck and Upper Extremities: A Proposal” by Inger Arvidsson *et al*. (2021)

**DOI:** 10.1093/annweh/wxab075

**Published:** 2021-09-01

**Authors:** Mikael Forsman, Xuelong Fan, Ida-Märta Rhén, Carl Mikael Lind

**Affiliations:** 1 Division of Ergonomics, School of Engineering Sciences in Chemistry, Biotechnology and Health, KTH Royal Institute of Technology, Hälsovägen 11C, SE-14157 Huddinge, Sweden; 2 IMM Institute of Environmental Medicine, Karolinska Institutet, SE-171 77 Stockholm, Sweden; 3 Centre for Occupational and Environmental Medicine, Stockholm County Council, SE-113 65 Stockholm, Sweden; 4 Department of Industrial and Materials Science, Chalmers University of Technology, SE-412 96 Gothenburg, Sweden

The recent article by Arvidsson *et al.* (2021) gives recommendations for so-called ‘action levels’ to be used for risk assessments based on technical measurements. It is an important article, since technical measurements are more accurate than those obtained by observational methods, and since technical measurement instruments now are becoming less expensive and more practically useful to quantify workload for both researchers and practitioners. Today, accelerometers used as inclinometers for measurement of postures and movements are often replaced by so-called inertia measurement units (IMUs), which in addition to accelerometers include gyroscopes. IMUs have shown high accuracy for upper arm postures and movements when compared to optical (gold standard) methods (Yang *et al.*, 2017; Chen *et al.*, 2018). These studies also confirmed the effect of the intrinsic errors of accelerometer in upper arm measurements, which Bernmark and Wiktorin (2002) showed, and explained as induced by the centripetal force acting on the accelerometers especially at high arm movements.

Arvidsson *et al*. represent a research group with an impressive publication history; they have developed methods and for about three decades carried out measurements in about 60 different occupation groups—with simultaneous standardized clinical health examinations (Balogh *et al.*, 2019). They have been consistent in their methods, low-pass filter cut-off frequencies, and output variables, and algorithms for computing variables have been kept the same throughout the decades. In the referred article, they wrote: ‘By inclinometry, we recorded work postures of the head, upper back, and upper arms…’, and ‘Movement velocities were then calculated by derivation’. It may lead the reader to interpret that arm velocity was computed by derivation of the arm elevation angle in similarity with wrist movement velocity, which is also included in their list of action levels and has been computed by derivation of the wrist flexion angle. However, the authors do refer to Hansson *et al.* (2006), where the arm velocity is computed as the so-called ‘generalized angular velocity’. The generalized angular velocity includes all three axes of the accelerometer and the velocity is the normalized resultant angular movement on the unit sphere per time unit. Since this also includes the radial axial movement, it is, in field measurements, higher than the inclination velocity.

We recently performed a study (Fan *et al.*, 2021), where we investigated the effect of sensor type, i.e. accelerometers versus IMUs, and the effect of computational arm velocity method, i.e., inclination versus generalized angular velocity, on postures and movements of the arm and trunk. To compute the upper arm posture percentiles from accelerometers, we followed the computational procedure as described by Hansson *et al.* (2006). These measures were compared with the percentiles from IMUs computed with the Kalman filter recommended by Chen *et al.*, 2018. Measurements from half a workday from 38 warehouse workers (men and women) were included. The group mean percentiles from the two sensor types were very similar. The differences were less than 2°, which indicate that percentiles from studies of different sensor types can be directly compared.

Then we compared the upper arm velocity percentiles from accelerometers only, with the velocity computed from accelerometers combined with gyroscopes. As for the postures, we also evaluated the influence of both sensor types and computational velocity method (i.e. inclination versus generalized arm angular velocity). The group mean percentiles from the two sensor types and the two different computational methods showed large differences. For the median angular velocities, accelerometers only showed about double as high velocities as those from accelerometers with gyroscopes, and the generalized velocity was about double as high as the inclination velocity. When combining the two factors, the mean median generalized velocity, from accelerometers only, was about 4.5 times as high as the mean median inclination velocity, from accelerometers with gyroscopes.

To find what the 60°/s action level of Arvidsson *et al.* (2021) corresponds to in the other three velocity distributions, we used the four velocity distributions of each worker and computed the corresponding action levels. The generalized velocity of 60°/s, with accelerometers only, corresponded to the inclination velocity of 27°/s from accelerometers only, to 26°/s in generalized velocity of from accelerometers with gyroscopes and to the inclination velocity of 13°/s from accelerometers with gyroscopes (Fan *et al.*, 2021). These values are obtained from a field study including one occupational group. Therefore, the exact corresponding velocities may be somewhat different for other occupational groups.

To conclude, while posture percentiles may be compared, it is clear that upper arm velocities depend greatly on sensor types and on chosen computational method, and that the recommended action level by Arvidsson *et al.* (2021) need to be transformed, with above given corresponding velocities or with at the time more recent ones, before being applied to measurements from IMUs and/or for inclination velocities, which have been used in several recent studies (see Fan *et al.*, 2021).
